# The effect of periodic ketogenic diet on newly diagnosed overweight or obese patients with type 2 diabetes

**DOI:** 10.1186/s12902-022-00947-2

**Published:** 2022-02-03

**Authors:** Sumei Li, Guoxin Lin, Jinxing Chen, Zhenxin Chen, Feipeng Xu, Feng Zhu, Jintian Zhang, Shouping Yuan

**Affiliations:** 1 Department o f Endocrinology, Teaching Hospital, The First Hospital of Putian, Fujian Medical University, Putian, Fujian Province People’s Republic of China; 2 Department of Pathology, Putian University, Medical University, Fujian, China

**Keywords:** Ketogenic diet, Obesity, Being overweight. Type 2 diabetes, Newly diagnosis

## Abstract

**Background:**

The ketogenic diet (KD) is characterized by fat as a substitute of carbohydrates for the primary energy source. There is a large number of overweight or obese people with type 2 diabetes mellitus (T2DM), while this study aims to observe periodic ketogenic diet for effect on overweight or obese patients newly diagnosed as T2DM.

**Methods:**

A total of 60 overweight or obese patients newly diagnosed as T2DM were randomized into two groups: KD group, which was given ketogenic diet, and control group, which was given routine diet for diabetes, 30 cases in each group. Both dietary patterns lasted 12 weeks, and during the period, the blood glucose, blood lipid, body weight, insulin, and uric acid before and after intervention, as well as the significance for relevant changes, were observed.

**Results:**

For both groups, the weight, BMI(body mass index), Waist, TG (triglyceride), TC(cholesterol), LDL (low-density lipoprotein cholesterol), HDL (high-density lipoprotein cholesterol), FBG (fasting glucose), FINS (fasting insulin), HbA1c (glycosylated hemoglobin) were decreased after intervention (P < 0.05), while the decrease rates in the KD group was more significant than the control group. However, UA(serum uric acid) in the KD group showed an upward trend, while in the control group was not changed significantly (P > 0.05).The willingness to adhere to the ketogenic diet over the long term was weaker than to the routine diet for diabetes.

**Conclusion:**

Among the overweight or obese patients newly diagnosed as type 2 diabetes mellitus, periodic ketogenic diet can not only control the body weight, but also control blood glucose and lipid, but long-term persistence is difficult.

## Background

By 2013, the prevalence rate of diabetes among Chinese people aged 18 or above had been as high as 10.4% [1]. At the same time, the number of obese people is increasing year by year. An epidemiological investigation in China showed that among obese people, the higher the body mass index, the higher the prevalence rate of type 2 diabetes [2]. According to relevant data, extremely low carbohydrate [3–9], adequate sleep, and appropriate exercise can control the blood glucose and lower the body weight loss among T2DM patients. The ketogenic diet (KD) pattern is of high fat, low carbohydrates, and appropriate protein. Characterized by fat as a substitute of carbohydrates for the primary energy source, KD was first used to treat refractory epilepsy in children [10]. In recent years, relevant scholars have found that this diet pattern may control blood glucose and lower body weight, and the purpose of this study is to observe the efficacy of periodic ketogenic diet in overweight or obese patients newly diagnosed as T2DM.

## Methods

### General Information

A total of 60 overweight or obese patients newly diagnosed as T2DM in the Outpatient Service of Endocrinology Department in our hospital between June 1, 2018 and June 1, 2020 were included.To ensure the acceptability and compliance of the study diet, the enrolled patients were required to adhere to the diet during the study. The enrolled patients were invited to participate in three face-to-face communication sessions before the study, and participated in the nutrition knowledge popularization training. The aim is to remove the patient’s negative concerns and gain support from family members. All of them signed the informed consent form. They were randomized into two groups: KD group, which was given ketogenic diet, and diabetes diet control group, which was given routine diet for diabetes, 30 cases in each group. Both dietary patterns lasted 12 weeks, and during the period, relevant indicators before and after intervention, as well as the significance for relevant changes, were observed. Inclusion criteria: Patients aged 18 to 50 years, BMI≥25 kg/m2, newly diagnosed as T2DM, without medication history of hypoglycemic agent, and HbA1c < 10%. Exclusion criteria: Patients who had complicated with serious heart, liver, lung, kidney, or brain disease, or history of serious acute or chronic complications for diabetes, those who underwent infection, pregnancy, trauma, or surgery, and pregnant or lactating women, and those who used drugs that may cause glucose metabolism disorders.

### Methods

The 60 patients were randomized into two groups: KD group, which was given ketogenic diet, and diabetes diet control group, which was given routine diet for diabetes. For the KD group, the main foods for the diet were olive oil, butter, fried eggs, double-fried pork, pan-fried salmon, pacific saury, sardines, broccoli, avocado, and so on, and daily limits for ingredients were as follows: carbohydrate 30-50 g, protein 60 g, fat 130 g, and total calories (1500±50) Kcal. For the control group, foods were not limited, and daily limits for ingredients were as follows: carbohydrate 250-280 g, protein 60 g, fat 20 g, total calories (1500 ±50) Kcal. For both groups, each subject should consume more than 2000ml of water every day during the diet control period. For the included cases, relevant data at baseline and 12 weeks after intervention were evaluated, and FBG and FINS were determined. Their height, weight and waist circumference were measured, and body mass index (BMI) was calculated. At the same time, HbA1c, UA, TC, LDL-C, HDL-C and TG were tested. All of the subjects received a 12-week dietary intervention. The person-times of hypoglycemia during this period were recorded. Symptoms of hypoglycemia: hunger, cold sweat, palpitations, hand tremors, and fatigue. Hypoglycemia event: Blood glucose < 3.9 mmol/L.

### Statistical Analysis

SPSS 22.0 software was used for statistics, and the results were presented. T-test or rank sum test was used for data comparison between and within groups, and chi-square test was used for rate comparison. *P* < 0.05 was considered statistically significant.

## Results

### Comparison of general information before intervention

Before intervention, there were no statistically significant differences between the two groups in gender, age and course of disease, and in Weight, BMI, Waist, TG, TC, LDL, HDL, FBG, FINS, HbA1c, and UA as well (P > 0.05), as shown in Table 1.

**Table 1:** Comparison of general data between the two groups before intervention ($\overline x\pm s$)

project	The ketogenic diet group	The diabetic diet group	P
Age (yr)	36.50±13.67	37.10±14.02	0.657
Course(month)	3.51±1.40	3.42±1.38	0.537
Waist(cm)	108.53±12.13	107.33±12.07	0.712
Weight (kg)	78.32±15.27	77.95±14.76	0.854
BMI (kg/m2)	29.04±5.81	29.75±6.07	0.934
UA(umol/L)	378.23±24.35	381.42±26.64	0.669
HbA1C(%)	8.74±1.63	8.69±1.59	0.673
FBG (mmol/L)	9.01±2.77	8.98±2.48	0.940
FINS (pmol/L)	48.61±17.83	45.9±14.38	0.687
LDL (mmol/L)	2.75±0.65	2.77±0.69	0.864
HDL (mmol/L)	1.08±0.11	1.09±0.19	0.469
TG (mmol/L)	1.76±0.59	1.81±0.78	0.717
TC (mmol/L)	4.54±0.69	4.56±0.67	0.830

### Changes of indicators for both groups before and after intervention (Table 2)

After 12 weeks, 6 patients in the KD group withdrew from the study, for they could not adhere to the diet, i.e., 24 cases completed the study. In the control group, 1 case withdrew and 29 completed the study. For both groups, the Weight, BMI, Waist, TG, TC, LDL, HDL, FBG, FINS and HbA1c were decreased after intervention (*P* < 0.05). The decrease rates of body mass, blood lipid and blood glucose in the KD group was significantly higher than in the control group (*P* < 0.05).The UA in the KD group showed an upward trend, while for the UA change after intervention in the control group, there was no statistical significance (*P* > 0.05).

### Blood glucose

During Weeks 1-4 of intervention, 10 person-times of hypoglycemia symptoms and 2 person-times of hypoglycemia events (peripheral blood glucose < 3.9 mmol/L) occurred in the KD group, while 2 person-times of hypoglycemic symptoms and 0 person-time of hypoglycemic events (peripheral blood glucose < 3.9 mmol/L) occurred in the control group. No hypoglycemia symptoms or hypoglycemia events occurred during Weeks 5-12 of intervention. At the end of the intervention, 9 patients in the KD group had normal blood glucose, while 2 patients in the control group had normal blood glucose.

**Table 2:** Changes of indicators before and after intervention of different dietary patterns in the two groups()

project	The ketogenic diet group(*n* = 24)		P	The diabetic diet group(*n* = 29)		P
	Before the intervention	After 12 weeks of intervention		Before the intervention	After 12 weeks of intervention	
Waist(cm)	108.53±12.13	99.24±14.58	0.000	107.33±12.07	106.56±9.78	0.000
Weight (kg)	78.32±15.27	70.26±14.79	0.000	77.95±14.76	77.34±13.28	0.000
BMI (kg/m2)	29.04±5.81	26.21±5.74	0.000	29.75±6.07	29.42±5.97	0.000
HbA1C(%)	8.74±1.63	7.82±1.43	0.000	8.69±1.59	8.42±1.51	0.000
FBG (mmol/L)	9.01±2.77	7.62±1.69	0.000	8.98±2.48	8.42±2.17	0.000
FINS (pmol/L)	88.61±17.83	40.38±9.54	0.000	87.91±14.38	84.21±10.79	0.000
LDL (mmol/L)	2.75±0.65	2.34±0.45	0.018	2.77±0.69	2.59±0.58	0.139
HDL (mmol/L)	1.08±0.11	1.21±0.23	0.000	1.09±0.19	1.12±0.20	0.000
TG (mmol/L)	1.76±0.59	1.44±0.26	0.000	1.81±0.78	1.66±0.46	0.000
TC (mmol/L)	4.54±0.69	4.02±0.43	0.000	4.56±0.67	4.23±0.47	0.000
UA (umol/L)	378.23±24.35	467.43±35.67	0.000	381.42±26.64	378.54±25.79	0.237

### Follow up for willingness to adhere

After the study, a follow up for willingness to adhere to the diet patterns was conducted. The results showed that the willingness in the KD group was lower than in the diabetes diet control group. Most patients reckoned that foods deficient in carbohydrates were unpleasant. The results are as shown in Table 3.

**Table 3:** Differences in intentions after completion of projects between the two groups

	The ketogenic diet group	The diabetic diet group	p
Number of people who cannot stick to it (percentage)	6(20%)	1(3.3%)	<0.05
Number of People willing to stick with it in the short term (proportion)	19(63.3%)	5(16.7%)	<0.05
Number of people willing to stick with it for the long term (proportion)	5 (16.7%)	24 (80%)	<0.05

## Discussion

The incidence rate of T2DM is increasing year by year. The main environmental factors for T2DM include high calorie diet, obesity, physical inactivity and etc. Worldwide, not only the prevalence of obesity has raised morbidity and mortality for cardiovascular and cerebrovascular diseases, diabetes, and cancers [11], but also has brought about huge expenses in healthcare. Therefore, it is important to effectively control obesity for reducing or saving relevant medical expenses [11, 12]. A relevant study [13] showed that proper daily exercise and dietary intervention not only caused effective weight loss, but also lowered the incidence of T2DM, thus reducing the risks of all-cause mortality and cardiovascular mortality. In this study, the overweight or obese patients initially diagnosed with T2DM were given 12 weeks of KD intervention before the application of hypoglycemic agents, and the changes of relevant indicators, e.g., blood glucose, blood lipid, body weight, uric acid, and insulin resistance, were observed.

The KD pattern had been often questioned by scholars for its high fat and extremely low carbohydrate until 2017, when a PURE study was published in the Lancet [14]. The study suggested that excessive carbohydrate intake was associated with the increase of total mortality. Since then, scholars began to reevaluate the value of KD. KD is a pattern deduced by people through theoretical research. As a therapeutic dietary pattern, it resulted from accumulation of large amounts of scientific knowledge, and so, it is of practicability with certain theoretical advantages [15].

KD with low carbohydrate content may simulate the state of starvation in the body, forming hunger ketosis. Thus, for the energy supply pattern of the body, the energy supply mode based on glucose was replaced by that based on ketone body, which requires fat to promote catabolism and reduce fat synthesis, while gluconeogenesis increases energy consumption. For this, the insoluble triglyceride is transformed into a water soluble ketone body (acetoacetate, β- hydroxybutyric acid soluble in water, and acetone insoluble in water). Therefore, The ketone body can be further excreted through the excretion of urine, carrying away energy [16]. In addition, a rise in the ketone body can suppress appetite [17], and so, the principle of the KD for weight loss is from many aspects [18].This may also explain why KD can decrease lipid metabolism indexes, e.g., triglyceride, total cholesterol, and low density lipoprotein though with high fat. This study showed that for both groups, after limiting calories in diet, the Weight, BMI, Waist, TG, TC, LDL, HDL, FBG, FINS, and HbA1c decreased (*P* < 0.05). In the KD group, different degrees of starvation were simulated, and ketone body became an important way for energy supply to the body. Therefore, the decrease rates of body mass, blood lipid and blood glucose in the KD group were significantly higher than in the control group.

KD emphasizes extremely low carbohydrate intake, which can affect the basic metabolism of sugar through regulating the decomposition rate of liver glycogen, thus reducing the blood glucose [19]. KD may reduce the absorption of intestinal monosaccharides, lower the blood glucose and alleviate the blood glucose fluctuation. A Goday et al. [20] confirmed the safety, tolerance, and effectiveness of short-term KD among the patients with T2DM.

A study of Myette. Cote et al. [21] has verified that KD can rapidly and significantly improve the patients’ blood glucose control, thus lowering the level of feedback fasting insulin level, stabilizing the blood glucose and alleviating the blood glucose fluctuation in patients with T2DM. Laura R Saslow et al. [22] also achieved good efffects in controlling blood glucose and body weight through an online intervention in the diet of overweight T2DM patients. The study of Partsalaki I et al. [23] has shown that KD can reduce waist circumference, body weight and insulin resistance as well. The waist circumference is an important indicator of central obesity, and a factor related to insulin resistance as well. This study showed that with the decrease of waist circumference, the body mass was decreased, blood glucose was controlled, the insulin resistance was alleviated, and related lipid metabolism indexes of the subjects were improved. The body mass reduction was closely related to the adoption of KD pattern and the negative nitrogen balance caused by calorific restriction. Therefore, all related indexes were improved in the control group of relatively low calorie. The individuals should have a relatively low caloric intake, or even the application of KD cannot significantly improve the body composition [24].The existing studies have primarily demonstrated the effects of KD in blood glucose improvement and body weight loss, but it was difficult to recover the blood glucose to normal because the selected patients with diabetes had a long course of disease and more obviously impaired islet function. The subjects in this study were overweight or obese patients newly diagnosed as T2DM. For the patients newly diagnosed as T2DM, the insulin resistance is often significant, and the islet function declines to some extent, but the impairment of islet function is not so serious. KD can significantly alleviate the insulin resistance, and at the same time, it may reduce body weight and fat. Thus, the blood glucose control may be more reliable. The innovative point for this study consists in the newly diagnosed overweight or obese patients without medication for blood glucose control, some of whom had blood glucose under control through KD regulation, a change in diet without medication. For some patients with diabetes, this will be greatly different. However, the observation for this study only lasted 12 weeks, which was not enough to clarify the recurrence of hyperglycemia after discontinuation of KD.After patients with type 2 diabetes discontinue the periodic ketogenic diet, blood glucose may continue to be well controlled in some patients, and blood glucose may rise in others. This requires further and longer follow-up studies. At the same time, it should be noted that hypoglycemia events occurred during the KD period, especially during the first 4 weeks. Although all of the patients were tolerant in the later stage, we should still pay attention to this. In addition, the inevitable serum uric acid increase accompanied with KD cannot be ignored because it may increase the risk of gout attacks. Therefore, during the intervention, it is necessary to drink enough water for promoting uric acid excretion, and as appropriate, sodium bicarbonate may be given to alkalize the urine, thus facilitating the excretion of uric acid, and reducing the risk of hyperuricemia. Admittedly, although KD may impact weight loss and T2DM greatly, it is unpleasant for extremely low carbohydrate. Therefore, long term adherence to KD in daily life is difficult for most people. At present, it is merely a short-term diet pattern for relevant treatment.

## Conclusions

The periodic ketogenic diet can control not only weight but also blood glucose and blood lipid in patients with overweight or obese T2DM. But long-term persistence is difficult. It can be a therapeutic model of diet. Some newly diagnosed overweight or obese people with type 2 diabetes may benefit from weight loss, and some patients may be able to achieve good blood glucose control in a short term without medication.

## Data Availability

The datasets used in the analyses described in this study are available from the corresponding author on reasonable request.

## Abbreviations

KD: Ketogenic diet
FBG: Fasting glucose
FINS: Fasting insulin
BMI: Body mass index
HbA1c: Glycosylated hemoglobin
UA: Serum uric acid
TC: Cholesterol
LDL-C: Low-density lipoprotein cholesterol
HDL-C: High-density lipoprotein cholesterol
TG: Triglyceride

## References

[1] Wang L, Gao P, Zhang M, Huang Z, Zhang D, Deng Q (2017). Prevalence and ethnic pattern of diabetes and prediabetes in China in 2013. JAMA.

[2] GBD 2015 Obesity Collaborators. Afshin A, Forouzanfar MH, Reitsma MB, Sur P, Estep K, et al. Health Effects of Overweight and Obesity in 195 Countries over 25 Years. N Engl J Med. 2017;377(1):13–27. doi: 10.1056/NEJMoa1614362. 10.1056/NEJMoa1614362 PMC5477817 28604169

[3] Ruskin DN, Masino SA (2012). The nervous system and metabolic dysregulation: emerging evidence converges on ketogenic diet therapy. Front Neurosci.

[4] Dashti HM, Mathew TC, Khadada M, Al-Mousawi M, Talib H, Asfar SK, et al. Beneficial effects of ketogenic diet in obese diabetic subjects. Mol Cell Biochem. 2007;302(1-2):249-256. doi: 10.1007/s11010-007-9448-z. 10.1007/s11010-007-9448-z 17447017

[5] Feinman RD, Pogozelski WK, Astrup A, Bernstein RK, Fine EJ, Westman EC (2015). Dietary carbohydrate restriction as the first approach in diabetes management: critical review and evidence base. Nutrition.

[6] Bistrian BR, Blackburn GL, Flatt JP, Sizer J, Scrimshaw NS, Sherman M (1976). Nitrogen metabolism and insulin requirements in obese diabetic adults on a protein-sparing modified fast. Diabetes.

[7] Volek JS, Phinney SD, Forsythe CE, Quann EE, Wood RJ, Puglisi MJ (2009). Carbohydrate restriction has a more favorable impact on the metabolic syndrome than a low fat diet. Lipids.

[8] Kirk JK, Graves DE, Craven TE, Lipkin EW, Austin M, Margolis KL (2008). Restricted-carbohydrate diets in patients with type 2 diabetes: a meta-analysis. J Am Diet Assoc.

[9] Accurso A, Bernstein RK, Dahlqvist A, Draznin B, Feinman RD, Fine EJ (2008). Dietary carbohydrate restriction in type 2 diabetes mellitus and metabolic syndrome: time for a critical appraisal. Nutr Metab (Lond).

[10] Caraballo R, Noli D, Cachia P (2015). Epilepsy of infancy with migrating focal seizures: three patients treated with the ketogenic diet. Epileptic Disord.

[11] Cole CB, Nikpay M, Stewart AF, McPherson R (2016). Increased genetic risk for obesity in premature coronary artery disease. Eur J Hum Genet.

[12] Williams EP, Mesidor M, Winters K, Dubbert PM, Wyatt SB (2015). Overweight and Obesity: Prevalence, Consequences, and Causes of a Growing Public Health Problem. Curr Obes Rep.

[13] Lindström J, Ilanne-Parikka P, Peltonen M, Aunola S, Eriksson JG, Hemiö K (2006). Sustained reduction in the incidence of type 2 diabetes by lifestyle intervention: follow-up of the Finnish Diabetes Prevention Study. Lancet.

[14] Dehghan M, Mente A, Zhang X, Swaminathan S, Li W, Mohan V (2017). Associations of fats and carbohydrate intake with cardiovascular disease and mortality in 18 countries from five continents (PURE): a prospective cohort study. Lancet.

[15] Caprio M, Infante M, Moriconi E, Armani A, Fabbri A, Mantovani G (2019). Very-low-calorie ketogenic diet (VLCKD) in the management of metabolic diseases: systematic review and consensus statement from the Italian Society of Endocrinology (SIE). J Endocrinol Invest.

[16] Urbain P, Bertz H (2016). Monitoring for compliance with a ketogenic diet: what is the best time of day to test for urinary ketosis?. Nutr Metab (Lond).

[17] Nymo S, Coutinho SR, Jørgensen J, Rehfeld JF, Truby H, Kulseng B (2017). Timeline of changes in appetite during weight loss with a ketogenic diet. Int J Obes (Lond).

[18] Paoli A, Rubini A, Volek JS, Grimaldi KA (2013). Beyond weight loss: a review of the therapeutic uses of very-low-carbohydrate (ketogenic) diets. Eur J Clin Nutr.

[19] American Diabetes Association (2013). Standards of medical care in diabetes--2013. Diabetes Care.

[20] Goday A, Bellido D, Sajoux I, Crujeiras AB, Burguera B, García-Luna PP (2016). Short-term safety, tolerability and efficacy of a very low-calorie-ketogenic diet interventional weight loss program versus hypocaloric diet in patients with type 2 diabetes mellitus. Nutr Diabetes.

[21] Myette-Côté É, Durrer C, Neudorf H, Bammert TD, Botezelli JD, Johnson JD (2018). The effect of a short-term low-carbohydrate, high-fat diet with or without postmeal walks on glycemic control and inflammation in type 2 diabetes: a randomized trial. Am J Physiol Regul Integr Comp Physiol.

[22] Saslow LR, Mason AE, Kim S, Goldman V, Ploutz-Snyder R, Bayandorian H (2017). An online intervention comparing a very low-carbohydrate ketogenic diet and lifestyle recommendations versus a plate method diet in overweight individuals with type 2 diabetes: A randomized controlled trial. J Med Internet Res.

[23] Partsalaki I, Karvela A, Spiliotis BE. Metabolic impact of a ketogenic diet compared to a hypocaloric diet in obese children and adolescents. J Pediatr Endocrinol Metab. 2012;25(7–8):697–704. 10.1515/jpem-2012-0131. 10.1515/jpem-2012-0131 23155696

[24] Hall KD, Chen KY, Guo J, Lam YY, Leibel RL, Mayer LE (2016). Energy expenditure and body composition changes after an isocaloric ketogenic diet in overweight and obese men. Am J Clin Nutr.

